# Isolation of nitrate-reducing bacteria from an offshore reservoir and the associated biosurfactant production†

**DOI:** 10.1039/c8ra03377c

**Published:** 2018-07-25

**Authors:** Fuqiang Fan, Baiyu Zhang, Penny L. Morrill, Tahir Husain

**Affiliations:** Northern Region Persistent Organic Pollution Control (NRPOP) Laboratory, Faculty of Engineering and Applied Science, Memorial University of Newfoundland St. John's NL Canada A1B 3X5 ff0466@mun.ca bzhang@mun.ca thusain@mun.ca; Earth Sciences, Faculty of Science, Memorial University of Newfoundland St. John's NL Canada A1B 3X5 pmorrill@mun.ca

## Abstract

Biosurfactant producing nitrate-reducing bacteria (NRB) in anaerobic reservoir environments are closely associated with souring (H_2_S) control in the offshore oil and gas industry. Five NRB strains were screened from offshore produced water samples and all were identified as *Pseudomonas stutzeri*. Their biosurfactant producing abilities when fed on either glucose or glycerol media were investigated. *P. stutzeri* CX3 reduced the medium surface tension to 33.5 and 29.6 mN m^−1^, respectively, while growing on glucose or glycerol media. The CX3 strain was further inoculated to examine its growth performance, resulting in 32.4% and 94.5% of nitrate consumption over 228 hours of monitoring in two media, respectively. The composition analysis of the biosurfactant product generated by *P. stutzeri* CX3 was conducted through thin-layer chromatography, gas chromatography with a flame ionization detector (FID) and Fourier transform infrared spectroscopy (FT-IR). The biosurfactant product was identified as a mixture of a small part of lipopeptides and a large part of glycolipids while its critical micellar concentration (CMC) was as low as 35 mg L^−1^. The biosurfactant product demonstrated high stability over a wide range of temperature (4–121 °C), pH (2–10), and salinity (0–20% w/v) concentration. The results provided valuable technical and methodological support for effective offshore reservoir souring control and associated enhanced oil recovery activities.

## Abstract 2

Biosurfactant producing nitrate-reducing bacteria (NRB) in anaerobic reservoir environments are closely associated with souring (H_2_S) control in the offshore oil and gas industry.

## Graphical abstract

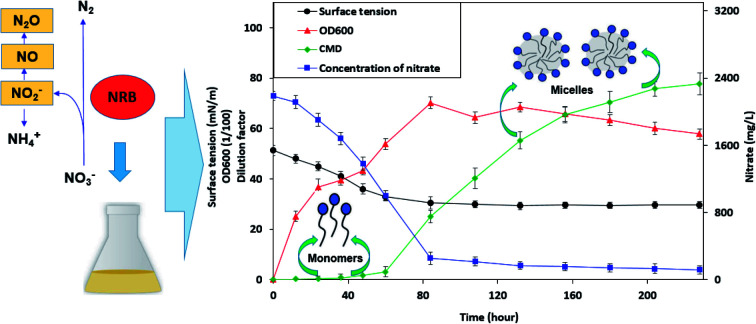

## 1. Introduction

The activity of sulfate reducing bacteria (SRB) has long been a major concern in oilfield water systems and offshore petroleum reservoirs because these microorganisms are one of the main causes of reservoir souring (sulfides).^1^ The undesirable production of sulfides by SRB can result in reduced quality of produced oils, health and safety risks for operators, and increased corrosivity of produced fluids.^2,3^

Although sulfides can be removed chemically after their production, *in situ* elimination through continuous nitrate/nitrite injection has also proven to be effective, as demonstrated both in model column,^4–6^ inland^7,8^ and offshore field studies.^9,10^ Nitrate injection changes the microbial community in the subsurface from mainly SRB to one enriched in NRB, which include the nitrate-reducing, sulfide-oxidizing bacteria (NR-SOB) that oxidize H_2_S directly and the heterotrophic NRB (hNRB) that compete with SRB for degradable organic electron donors. Additionally, both types of NRB also promote SRB inhibition *via* production of nitrite formed in nitrate reduction pathways.^11^ NRB are well known for their denitrifying capacity in which nitrates or nitrites are converted into nitrogen-containing gases. This function enables NRB to play significant roles in the global nitrogen cycle^12^ and mitigation and control of sulfide induced reservoir souring problems in offshore oil fields.^13^ However, understanding of the detailed microbial mechanisms involved in NRB-SRB interactions during nitrate/nitrite injections for offshore reservoirs souring mitigation is currently limited.

Anaerobic, indigenous NRB have the potential to produce specific biosurfactants in deep geological porous offshore reservoirs with diverse physiochemical *in situ* conditions. Biosurfactants are surface-active compounds with both lipophilic and hydrophilic structural moieties produced by microorganisms, which either adhere to the cell surface or are secreted extracellularly in the growth medium.^14^ These surface active molecules reduce surface tension at air–water interfaces and interfacial tension in both aqueous solutions and hydrocarbon mixtures.^15^ Notably, biosurfactants have various advantages over their commercially manufactured counterparts because of their lower toxicity, biodegradable nature, ease of biosynthesis and the effectiveness under extreme conditions such as temperature, pH, and salinity.^16,17^ Fallon *et al.*^18^ have confirmed that biosurfactants can be naturally derived from NRB. As microorganisms capable of utilizing hydrocarbons as carbon and energy sources, NRB will produce surface-active agents as by-products to facilitate hydrophobic degradation.^19^ The selective surface-active agents or biosurfactants produced by NRB increase the surface area of hydrophobic water-insoluble substrates (low molecular weight biosurfactants) and increase the solubility (high molecular weight biosurfactants), thus improving the bioavailability of hydrocarbons for NRB.^17^ When emulsion occurs close to the cell surface of NRB, each cluster of cells creates its own microenvironment and stimulate the growth of NRB in oil and gas reservoirs.^19^ This mechanism enables NRB to out-compete harmful SRB for basic carbon nutrients. The SRB will be inhibited from producing new hydrogen sulfide/iron sulfide, and the existing sulfides will be removed by bacterial degradation, resulting in effective control of offshore reservoir souring. Thus, in this point biosurfactants are interesting by-products involved in the SRB/NRB competition.

To investigate how biosurfactants affect NRB/SRB competition in a reservoir, successful screening of biosurfactant producing NRB and generation of associated biosurfactants are needed. Oil reservoirs could provide a unique hydrocarbon-rich environment for biosurfactant screening and the enrichment of diverse biosurfactant producers.^20^ The extreme conditions (*e.g.*, high temperature, high pressure, and low oxygen concentration) in oil reservoirs would formulate a microbial community that may be distinguished from others.^21^ So far, biosurfactant producers identified from oil reservoirs are mainly from inland reservoirs and limited to the genera of *Bacillus*^22–26^ and *Pseudomonas*.^27–29^ Currently, very limited marine biosurfactant producers from offshore oil and gas fields have been reported and it was unclear whether they were NRB species or not. Cai *et al.*^30^ isolated marine biosurfactant producers from crude oil, formation water, drilling mud, and treated produced water samples in offshore oil and gas fields. The genotype and phylogenetic relation of these isolates were investigated and biosurfactant producers were primarily found in the genera of *Rhodococcus* and *Halomonas*. However, most of these species reported are general biosurfactant producers and they were screened under aerobic conditions, which were not prevalent in the reducing reservoir environments. Hui *et al.*^31^ evaluated the microbial community structure and functionally distinct groups in three kinds of produced water samples from the shallow, mesothermic and low-salinity Daqing oil reservoir using both culture-dependent and culture-independent methods. The isolates affiliated to *Pseudomonas stutzeri* PTG4-15 (DP26, BP39, and PW5) were initially identified as nitrate-reducing bacteria, biosurfactant-producing bacteria, and polymer-producing bacteria. This indicates that anaerobic indigenous NRB have the potential to produce specific biosurfactants in the reservoir.

Until now, biosurfactant producing NRB isolated from oil reservoirs have been rarely reported, and associated biosurfactant production is extremely limited in the literature. No previous study tackled the isolation of biosurfactant producing NRB from offshore reservoirs and subsequent anaerobic biosurfactant production. Therefore, the aim of this study was to screen NRB strains from an offshore reservoir, and conduct biosurfactant production and characterization. The research outputs will not only help identify NRB and generate biosurfactants under anaerobic conditions, but also provide technical and methodological support for further identifying NRB/SRB interactions and generating methodologies for effective offshore reservoir souring control in the future.

## 2. Materials and methods

### 2.1 Source and collection of inoculum

Produced water samples in an offshore water flooding reservoir were collected for screening novel biosurfactant producers. Injection wells on the platforms were injected with nitrate/nitrite to stimulate the growth of indigenous nitrate-reducing microorganisms in the reservoir or exogenous NRB strains from seawater injection process. Produced water was collected in 1 liter sterile glass bottles and then sealed immediately to maintain anoxic conditions. The samples were stored in a refrigerator in darkness before shipping. Subsequently, the samples were packaged with frozen ice packs and transported to the laboratory for enrichments and bacterial isolations. The major constituents of the produced water include (in wt/vol) chloride, 4.0%; sodium, 2.5%; sulfate, 0.13%; calcium 0.12%; and bicarbonate, 0.076%. Water samples were stored at 4 °C and were taken for enrichment culture within one week of collection.

### 2.2 Isolation and identification of NRB

#### 2.2.1 Growth media

Coleville synthetic brine (CSB) medium was selected for the NRB culturing based on the properties of produced water and NRB growth requirement. The recipe was composed of (in 1 liter) NaCl, 7.0 g; MgSO_4_·7H_2_O, 0.68 g; CaCl_2_·2H_2_O, 0.24 g; NH_4_Cl, 0.02 g; KH_2_PO_4_, 0.027 g; NaC_2_H_3_O_2_·3H_2_O, 0.68 g; KNO_3_, 1.0 g; NaHCO_3_, 1.9 g; resazurin, 0.0001 g; and ND trace metals (0.5 mL H_2_SO_4_, 2.28 g MnSO_4_·H_2_O, 0.5 g ZnSO_4_·7H_2_O, 0.5 g H_3_BO_3_, 0.025 g CuSO_4_·5H_2_O, 0.025 g Na_2_MoO_4_·2H_2_O, and 0.045 g CoCl_2_·6H_2_O per liter), 50 mL per liter.^32^ The medium pH was then adjusted to between 7.0 and 7.5. After autoclaving, cooling, and equilibration of the medium with chamber gas overnight, sterilized 2.5% (w/v) Na_2_S·9H_2_O (final concentration of 0.02% (w/v)) was added to remove residual oxygen. Solid growth media were prepared by adding a certain amount of agar (2% w/v).

#### 2.2.2 Enrichment and isolation

Enrichments were prepared by adding 5 mL of produced water to 125 mL medium in sterile conical flasks under anaerobic conditions. To ensure the growth of microorganisms, three enrichment recipes with various carbon sources were prepared in conical flasks, respectively. The first one was adopted from Hui *et al.*^31^ and contained (in 1 liter) peptone, 20 g; beef extract, 10 g, KNO_3_, 1.0 g; NaCl, 0.7 g; KCl, 0.7 g; MgCl_2_·6H_2_O, 10 g; MgSO_4_·7H_2_O, 5.4 g; CaCl_2_·2H_2_O, 1.0 g. The second recipe was a raw screening medium which contained (in 1 liter) peptone, 5.0 g; beef extract, 3.0 g; KNO_3_, 1.0 g and trace metals (same as in CSB medium). The third recipe used citrate as the carbon source and contained l-asparagine 0.5 g; KNO_3_, 1.0 g; trisodium citrate, 0.85 g; MgSO_4_·7H_2_O, 0.5 g; FeCl_3_·6H_2_O, 0.05 g; CaCl_2_·2H_2_O, 0.24 g; and KH_2_PO_4_, 0.05 g. Trace metals were added following the trace element solution in CSB medium. All the media pHs were buffered between 7.2–7.5 and further treated with sterilized Na_2_S·9H_2_O.

Enrichment was initially conducted in a chamber filled with nitrogen until observable turbidity occurred. The bacterial consortia were then inoculated into new media for further acclimatization. The average bacterial acclimatization period was 15 days. After seven periods, 5 mL of each medium broth were transferred into fresh liquid CSB medium and the citrate medium. The second round of bacterial acclimatization in liquid CSB and citrate medium were conducted through three periods. After that, the consortia were serially diluted to 10^6^, 10^5^, and 10^4^ and then spread on solid CSB and citrate medium agar plate, respectively. The resulting plates were incubated at room temperature in a nitrogen-filled environment.

Routine growth and maintenance of broth isolates were in CSB medium. Bacterial growth status was detected by observing an increase in optical density (at 600 nm) or the cell dry weight filtered from medium broth.

#### 2.2.3 Identification and phylogenetic characterization of isolates

The purified isolates were then subjected to 16S ribosomal RNA sequencing and amplified with universal bacterial primers 27F (5′-AGA GTT TGA TYM TGG CTC AG-3′) and 16SR10 (5′-ACG GCT ACC TTG TTA CGA CT-3′). PCR amplification was prepared using 1 μL (5–10 ng) of each DNA sample in a 50 μL PCR mix containing 25 μL 2 × buffer, 4 μL of 25 mM dNTP, 0.5 μL of each primer (20 μM), 0.25 μL of 5 U μL^−1^ Taq DNA polymerase (Sigma-Aldrich, Canada). PCR was performed in procedures as follows: 5 min at 95 °C for initial denaturation, followed by 30 cycles of 20 s at 95 °C, 20 s at 55 °C, and 90 s at 72 °C, followed finally by 10 min at 72 °C for final extension. An aliquot of each culture was used for a DNA template in a polymerase chain reaction (PCR) using the primer pair. After gel electrophoresis confirmation of successful PCR reaction, PCR products were subjected to a clean-up process and measured by a NanoDrop spectrophotometer to determine the concentrations. Lastly, sequencing reactions with the last PCR products were conducted and measured with Applied Biosystems 3730 DNA Analyzer in Creait Network of Memorial University of Newfoundland. The obtained DNA sequences were aligned with previously published sequences from the GenBank database of National Center for Biotechnology Information (NCBI) using the Basic Local Alignment Search Tool (BLAST) program.

### 2.3 Screening of the NRB isolates for biosurfactant producers

#### 2.3.1 Biosurfactant producing media

According to the morphological properties of the strains on the agar plate and associated 16S rRNA results, 5 strains were selected for subsequent biosurfactant producer screening. Two media with glycerol and glucose as carbon sources were selected for anaerobic biosurfactant production, respectively. The first one modified from Zhao *et al.*^33^ contained (in 1 liter) glycerol, 46.6 g; NaNO_3_, 3.0 g; K_2_HPO_4_, 4.0 g; KH_2_PO_4_, 5.7 g; MgSO_4_·7H_2_O, 0.4 g; CaCl_2_·2H_2_O, 0.17 g; NaCl, 2 g; yeast extract, 2.7 g. The second one was adjusted from a previous medium in NRB screening medium and contained (in 1 liter) glucose, 10 g; NaNO_3_, 3.0 g; K_2_HPO_4_, 3.36 g; KH_2_PO_4_, 3.4 g; MgSO_4_·7H_2_O, 0.68 g; CaCl_2_·2H_2_O, 0.24 g; NaCl, 2 g; yeast extract, 3.0 g; FeCl_3,_ 0.05 g. Trace metals were also added into the two media as described previously. After autoclaving, cooling, and equilibration of the medium with chamber gas overnight, a sterilized Na_2_S solution was also added as mentioned above.

#### 2.3.2 Biomass determination

Each isolate was incubated using the same conditions as in biosurfactant producing procedure for 228 hours. Subsequently, 10 mL culture sample was filtered through a pre-weighed 0.22 μm filter membrane and washed 2 times with 10 mL distilled water. The filter membrane was then dried in the oven for 24 h and cooled down in desiccators before measuring the final weight. The biomass was determined as cell dry weight (g L^−1^).

#### 2.3.3 Parafilm test

A 25 μL aliquot of bacterial broth was added to the hydrophobic surface of a parafilm. The shape and the diameter of the droplet on the surface were inspected after 3 min. The negative control was prepared with 0.5 M phosphate buffer.^34^

#### 2.3.4 Drop collapsing test

Drops of the cell-free supernatant were placed on an oil-coated, solid surface. The polar water molecules are repelled from the hydrophobic surface with the absence of surfactants in the liquid and the drops remain stable whereas the droplet spread out slightly or even collapse with the presence of surfactants.^35^

#### 2.3.5 Emulsification activity assay

The emulsification activity (E24, eqn(1)) of the culture broth was determined by addition of 5 mL culture broth to 5 mL hexadecane or kerosene and vortexed for 2 min to create an optimum emulsion. Tests were performed in duplicate for quality assurance purpose and the results were expressed using the average of two measurements.

1 E24 = *H*_EL_/*H*_S_ × 100%

where *H*_EL_ is the height of the emulsion layer and *H*_S_ is the height of the total solution.

#### 2.3.6 Surface tension measurement

Culture samples were centrifuged at 10 000 rpm for 20 min to remove microbial cells and the supernatant was subject to surface activity measurements. Surface tension was determined with a surface tensiometer (DuNouy Tensiometer, Interfacial, CSC Scientific) at room temperature according to the ring method. The values reported were the mean of triplicate measurements.

### 2.4 Performance demonstration of the selected strain

According to the results, one isolate was selected to further demonstrate the performance of biosurfactant production. The two surfactant production media with glycerol and glucose as carbon sources were used to investigate the performance of biosurfactant production. The incubation was conducted at 30 °C while shaking at 200 rpm.

The isolate was firstly inoculated into the flask containing 20 mL fresh glucose medium and incubated using the above conditions for 48 h. Then a 200 μL aliquot of the culture broth was inoculated into each flask with 20 mL glycerol or glucose producing medium. The following two tests at time intervals of 0, 12, 24, 36, 48, 60, 84, 128, 156, 180, 204 and 228 h were sampled with the whole flask, respectively. In addition, to investigate the nitrate reduction effect of the isolate on biosurfactant production, initial NaNO_3_ concentrations at 1, 2, 3, 4 and 5 g L^−1^ were used on the glycerol producing medium. Samples were collected after 192 h.

The optical density (OD_600_) of samples was employed as the index of bacterial growth.^36^ Absorbance was measured at *λ* = 600 nm using a UV-Visible spectrophotometer. All culture media were shaken for 5 s to homogenize the media before OD_600_ determination. The absorbance of the freshly autoclaved medium was adjusted to 0 as the blank control. The measurement of OD_600_ was performed in triplicate by sampling three times.

To observe the nitrate consumption by NRB, nitrate concentration in the culture was determined using a two-wavelength approach.^37^ A rapid measurement of sample absorption at 220 nm (A220) was conducted in a quartz cuvette and organic matter interference was eliminated through the second measurement at 275 nm (A275). Nitrate concentration was determined from standard curves prepared with NaNO_3_ (0.2–8 mg L^−1^). Additionally, surface tension changes over the period were monitored using the methodology mentioned above. Critical micelle dilution (CMD) analysis was conducted to evaluate the concentration of biosurfactant product generated during the investigation of cultivation kinetics.

### 2.5 Biosurfactant production and characterization

#### 2.5.1 Extraction of the biosurfactant product

The procedures of biosurfactant extraction from the culture broth mainly followed the protocol according to Silva *et al.*^38^ after 228 hours of incubation of CX3 in glycerol medium. The culture broth was initially centrifuged at 11 000 rpm for 20 min to remove bacterial cells. The supernatant was then acidified to pH 2.0 with concentrated HCl (1 moL L^−1^) and kept at 4 °C overnight to reduce the rhamnolipid solubility. The biosurfactant product was further recovered through the addition of two volumes of chloroform : methanol (v/v, 2 : 1) mixture. After shaking the mixture for 2 h, the lower organic phase was collected and evaporated to dryness by a rotary evaporator. The final precipitate was collected.

#### 2.5.2 Determination of critical micellar concentrations (CMC)

CMC is defined as the surfactant concentration necessary to initiate micelle formation. The CMC of generated biosurfactant product was determined by plotting the surface tensions as a function of biosurfactant concentration and it was defined from the intercept of two straight lines extrapolated from the concentration-dependent and concentration-independent sections.^39^

#### 2.5.3 Thin-layer chromatography (TLC) analysis

TLC analysis was conducted to preliminarily characterize the purified biosurfactant product. Ten microliter biosurfactant solution at a concentration of 200 mg L^−1^ in methanol was applied to a 20 × 20 silica gel TLC plate (Sigma Aldrich). The biosurfactant product was separated using CHCl_3_ : CH_3_OH : H_2_O (70 : 10 : 0.5, v/v/v) as developing solvent system with different color developing reagents. For detection of lipopeptide biosurfactant, ninhydrin reagent (0.5 g ninhydrin in 100 mL anhydrous acetone) was sprayed on the dry plates and red spots were visualized after keeping the plate at 105 °C for 5 min. Anthrone reagent (1 g anthrone in 5 mL sulfuric acid mixed with 95 mL ethanol) was used to reveal the presence of glycolipid biosurfactant in yellow spots. Also, lipid content was further visualized by iodine chamber.

#### 2.5.4 Lipid class determination

Lipid class composition was determined using an Iatroscan Mark VI TLC with flame ionization detector (FID), silica coated Chromarods and a three-step development method.^40^ The lipid extracts were applied to the Chromarods and focused to a narrow band using 100% acetone. The first development system was hexane : diethyl ether : formic acid (99.95 : 1 : 00.05). The rods were developed for 25 minutes, removed from the system for 5 minutes and replaced for 20 minutes. The second development was for 40 minutes in hexane : diethyl ether : formic acid (79 : 20 : 1). The final development system had two steps, the first was 100% acetone for two 15 minute time periods, followed by two 10 minute periods in chloroform : methanol : chloroform-extracted water (5 : 4 : 1). Before each solvent system, the rods were dried in a constant humidity chamber. After each development system the rods were scanned in the Iatroscan and the data were collected using Peak Simple software (ver 3.67, SRI Inc). The Chromarods were calibrated using standards from Sigma Chemicals (Sigma Chemicals, St. Louis, Mo., USA).

#### 2.5.5 Fatty acid analysis

For all samples, lipid extracts were transesterified using sulfuric acid and methanol for 1 hour at 100 °C. The fatty acid methyl esters (FAMEs) developed from the extracts were analyzed on an HP 6890 gas chromatography (GC) system with FID and a 7683 autosampler. The GC column was a ZB wax+ (Phenomenex, U.S.A.). The column length was 30 m with an internal diameter of 0.32 mm. The column temperature began at 65 °C and held this temperature for 0.5 minutes. The temperature ramped to 195 °C at a rate of 40 °C min^−1^, held for 15 minutes then ramped to a final temperature of 220 °C at a rate of 2 °C min^−1^. This final temperature was held for 0.75 minutes. The carrier gas was hydrogen and flowed at a rate of 2 mL per minute. The injector temperature started at 150 °C and ramped to a final temperature of 250 °C at a rate of 120 °C per minute. The detector temperature stayed constant at 260 °C. Peaks were identified using retention times from standards purchased from Supelco, 37 component FAME mix (product number 47885-U), Bacterial acid methyl ester mix (product number 47080-U), PUFA 1 (product number 47033) and PUFA 3 (product number 47085-U). Chromatograms were integrated using the Varian Galaxie Chromatography Data System, version 1.9.3.2. A quantitative standard purchased from Nu-Chek Prep, Inc (product number GLC490) was used to check the GC column about every 300 samples (or once a month) to ensure that the areas returned were as expected.

#### 2.5.6 Fourier transform infrared spectroscopy (FT-IR) analysis

IR spectroscopy was used for structure analysis of the extracted biosurfactant product based on the oscillation patterns of chemical bonds at characteristic frequencies. The IR absorption spectrum of the dried biosurfactant product was measured on a Bruker Tensor 27 FT-IR using 16 scans over the range of 500–4000 cm^−1^ (KBr beamsplitter). The signals were collected in transmittance mode with a Zn–Se attenuated total reflectance (ATR) spectroscopy which is commonly used for rhamnolipid analysis.^41^

#### 2.5.7 Stability characterization

The effect of temperature, pH, and salinity on the surface activity of generated biosurfactant product was investigated by changing surrounding conditions.^42^ Generally, 1 CMC of biosurfactant solution was prepared and maintained at a constant temperature of 0, 20, 40, 60, 80, 100 °C for 120 min and cooled at room temperature to determine the thermal stability of the biosurfactant product. The pH influence on the biosurfactant activity was determined by adjusting the biosurfactant solutions in the range 2.0–12.0 using HCl (2 N) and NaOH (2 N) solutions, and the effect of salinity on the surface activity of the biosurfactant product was assessed by using various concentrations of sodium chloride (0.5–20% in w/v). In each case, the stability of the biosurfactant solution was evaluated by the change of surface tension values (1 CMC in all tests) and determined in triplicate.

## 3. Results and discussion

### 3.1 Phylogenetic analysis and morphological characteristics of NRB isolates

These isolates were incubated under anaerobic conditions with acetate as the carbon source and electron donor. Although the screening of biosurfactant producing NRB was performed under the presence of both nitrate and sulfate, only nitrate (*i.e.*, NaNO_3_) was the electron acceptor in NRB growth. The NRB groups have been noted for their special capability of performing effective denitrification. Far more energy is released during the reduction of nitrate than during that of sulfate,^43^ which also enables the NRB to utilize nitrate as preferential electron acceptor. The addition of MgSO_4_·7H_2_O was mainly used to provide mineral salts for bacterial growth and balance the sulfate levels.^44^ Most of the isolated NRB species were anaerobically slow-growing and mature colonies were formed around ten days on the CSB medium. As shown in Table 1, with sequences obtained by doing a BLAST search, a similarity of 99% or 100% to *Pseudomonas stutzeri* was found by the alignment of the 16S rRNA gene sequences of all the selected five NRB strains isolated from offshore produced water.

**Table 1:** Identification of the isolated possible biosurfactant producers

Isolate ID	Query cover	Identity	Species name with highest match	Liquid medium morphology	Agar plate morphology (shape/margin/elevation/surface texture/color)
CX1	100%	100%	*Pseudomonas stutzeri*	Turbidity	Circular/entire/convex/smooth/beige
CX3	100%	100%	*Pseudomonas stutzeri*	Turbidity	Circular/entire/convex/smooth/brown
HF5	100%	99%	*Pseudomonas stutzeri*	Flocculent	Circular/raised/convex/radiate/beige
HF6	100%	99%	*Pseudomonas stutzeri*	Turbidity	Circular/raised/convex/radiate/beige
FX8	100%	99%	*Pseudomonas stutzeri*	Flocculent	Circular/curled/convex/smooth/transparent

The morphology characteristics of isolates were summarized in Table 1. In general, all the species could be summed into two categories: the brown one with entire margins and the light color or transparent ones with raised or curled margins. Notably, some isolates, HF5 and FX6 formed flocculants in liquid biosurfactant producing medium. The components of the flocculants were studied extensively and were a mixture of polysaccharides, proteins, lipids, glycolipids and glycoproteins.^45^ Bioflocculants could be produced by various functional microorganisms and are biodegradable, environmentally friendly and harmlessness to humans. Microorganisms with high bioflocculant-producing ability thus can be utilized to produce bioflocculants and be used in industrial fields such as drinking and wastewater treatment, downstream processing, and fermentation processes.^46^ The flocculated cells were previously reported to be immobilized inside the reactor for a continuous fermentation system without cell separation and recycling units, and the yields from the reactor were clean broths for further ethanol production.^47^ Viewed from this perspective, the two strains have promising applications in batch, fed-batch or continuous fermentation reactors for industrial nitrate removal activities.

**Fig. 1:**
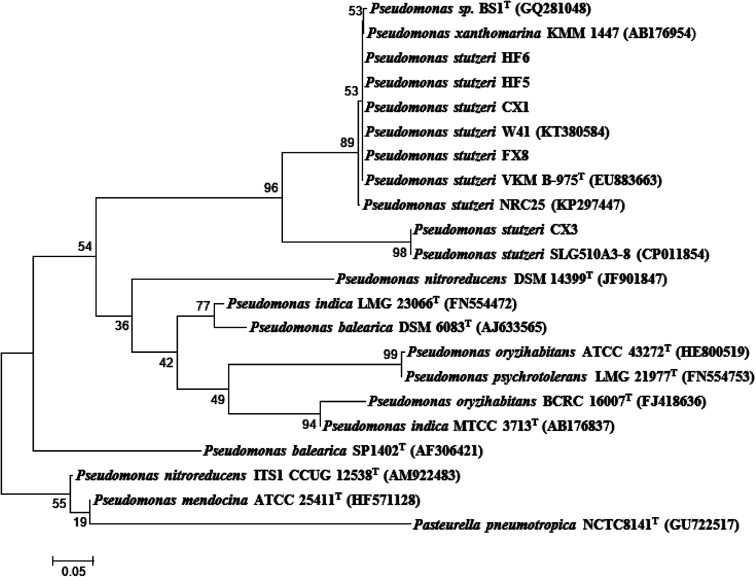
Phylogenetic tree of the isolated species from 16S rRNA gene sequences based on a neighbor-joining analysis of 1000 resampled datasets. The phylogenetic tree was constructed with MEGA 5.0 software based on the homologous 16S rDNA sequences. The GenBank accession numbers of reference species are listed in parentheses.

The strain CX3 was noticeable due to its different margin and color in the agar plate during morphology examination when compared with others (Table 1). During the growth on CSB medium, the isolate formed visible regular and glistening colonies with the colony diameters ranging from 1 to 3 mm. The precise taxonomic positions of the microbes were subsequently determined through the genotypic analysis on the basis of partial 16S rRNA sequencing. Unlike other four strains, *Pseudomonas stutzeri* CX3 occupied a unique branch in the phylogenetic tree generated from the five *Pseudomonas stutzeri* strains and other *Pseudomonas* species (Fig. 1). The selected *Pseudomonas stutzeri* strains formed a stable phyletic group within a heterogeneous cluster of *Pseudomonas xanthomarina*, and other *Pseudomonas stutzeri* strains, which distributed in ubiquitous environments and were identified among denitrifiers in natural materials.^48^*P. stutzeri* is a highly diverse species of great physiological and ecological versatility and is paid particular attention due to their specific activities in nitrification and denitrification processes.^49^ The strains of *Pseudomonas stutzeri* were also anaerobically isolated from various environmental samples and used in the degradation of aliphatic and aromatic hydrocarbons.^50,51^

### 3.2 Screening of biosurfactant-producing NRB

**Fig. 2:**
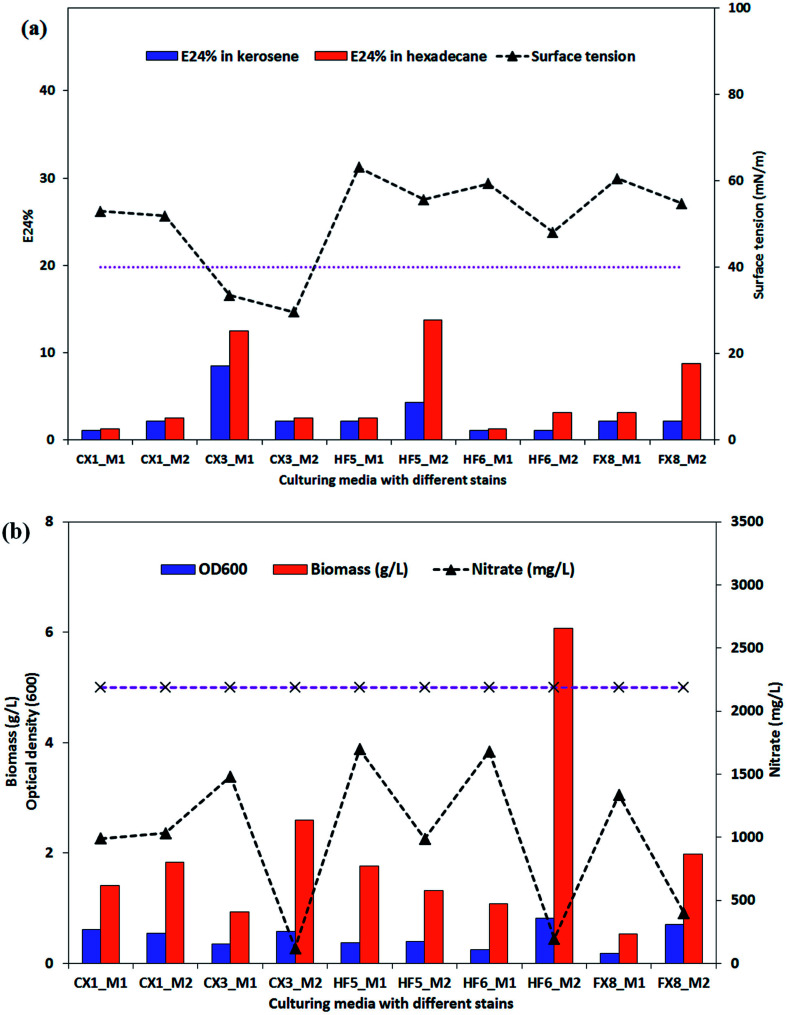
Evaluation parameters of the five *Pseudomonas stutzeri* strains in two growth media. Surface tension and E24% in kerosene and hexadecane were indicated in (a) while Biomass, OD_600_ and nitrate consumption were indicated in (b). All the tests were conducted in duplicate and the results were expressed as the average of two measurements. The analytical errors of the methodologies on all the parameters were below 7% and the accuracy was verified through 7 consecutive measurements of one sample.

After 228 hours of incubation, cell-free broths were subjected to a parafilm test for screening of potential biosurfactant producers. The results are presented in ESI Fig. S1† and it can be seen that the producing medium 2 yielded more flattened droplets for the *P. stutzeri* strains. The possible biosurfactant-producing strains were *P. stutzeri* CX3, CX1 and FX8. As indicated in Fig. 2a, the surface tension and E24% of the cell free culture of the 5 isolates on the two media were listed. It was observed that only the culture of *P. stutzeri* CX3 exhibited surface reducing ability on the glycerol and glucose media and lowered the surface tension to around 30 mN m^−1^ (the dotted line in Fig. 2a indicates the surface tension of 40 mN m^−1^). Consistent with the results from the parafilm test, all the isolates showed lower surface tension when incubated in the producing medium 2.

Although *P. stutzeri* HF5 presented the highest emulsification ability in hexadecane (13.8%) on producing medium 2, the NRB strains could not significantly emulsify *n*-hexadecane or kerosene. The biosurfactant-producing isolate CX3 showed very limited emulsification ability on production medium 2, which suggests that the capacity of biosurfactants for surface tension reduction was not necessarily correlated with emulsification capacity for forming and stabilizing emulsions. As surface active molecules, biosurfactants can form micelles at the interface of immiscible liquids by either reduction of surface and interfacial tension, or form stable emulsions between immiscible liquids.^52^ The former, which lowered surface and interfacial tensions, proved to be low-molecular-weight biosurfactants while the latter, which stabilized oil-in-water emulsions were more commonly high-molecular-weight ones and were referred as bioemulsifiers.^53^ The amphipathic biosurfactant product generated by isolate CX3 has limited emulsification potentials and are recognized as low-molecular-weight compounds.

The biomass production of the selected isolates and their nitrate consumption were also summarized in Fig. 2. The OD_600_ of broth samples was also used to evaluate the bacterial growth. As indicated by Fig. 2b, the OD_600_ values generally accorded with the biomass results, which was the measure of the dry weight of the bacterial cells (granular, flaky or flocculent) in the medium. For 4 of 5 strains, they consumed dramatically more nitrate when cultured on the producing medium 2, and among them CX3, HF6 and FX8 utilized 94.5%, 81.7% and 91.1% of the medium nitrate (the dotted line in Fig. 2b indicates the initial nitrate concentration) and yielded 2.6, 2.0 and 6.1g L^−1^ biomass by dry weight, respectively. This noticeable nitrate intake by microbes was attributed to nitrate respiration as the nitrate was utilized by bacteria as a terminal electron acceptor to maintain the redox balance (nitrate dissimilation) and as a nutrient (nitrate assimilation).^54^ Compared with other isolates, the extra nitrate intake in these three strains contributed to large bacterial growth while nitrate served as a nutrient (not electron acceptor) in nitrate assimilation in all media.

### 3.3 Biosurfactant production by NRB

**Fig. 3:**
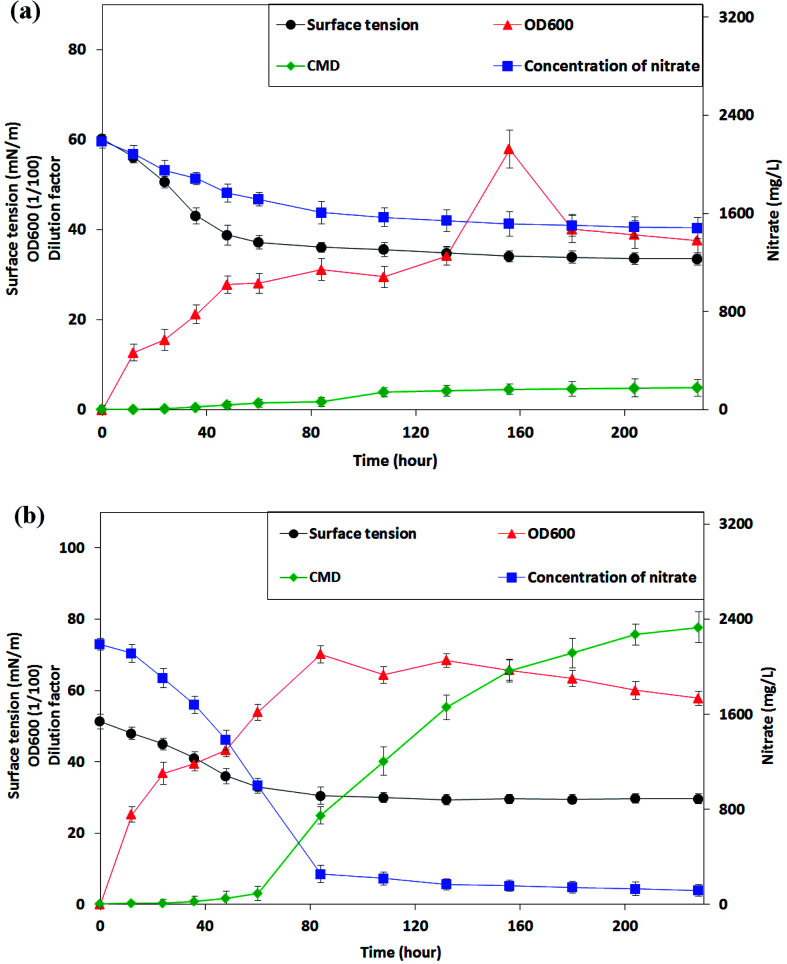
The changes of surface tension, cell growth and nitrate consumption of the *Pseudomonas stutzeri* CX3 on glucose (a) and glycerol (b) medium *versus* the incubation time.

From the phylogenetic results and the morphological characters of isolates, *P. stutzeri* CX3 exhibited differential performance when compared with other strains. Only *P. stutzeri* CX3, whose 16S rRNA sequence was deposited in Genbank under the accession number KY860630, showed significant surface-tension-lowering ability on the two biosurfactant producing media. As shown in Fig. 3a, the surface tension in culture broth gradually decreased to a plateau of around 34.0 mN m^−1^, while the nitrate concentration gradually decreased to a plateau of around 1500 mg L^−1^. The bacteria grew very fast during the initial 48 hours without a lag phase at a growth rate of 4.4 × 10^6^ CFU per mL per hour and reached their stationary phase after around 84 hours. The lack of adaption time in the bacterial growth may be due to the fact that NRB were pre-incubated for 48 h in the glucose medium. However, only 32.4% of the total nitrate in the medium was consumed over the study period.

CX3 grown on producing medium 2 grew more vigorously and a final nitrate consumption rate of 94.5% reached after 228 hours of incubation. From Fig. 3b, it can be seen that the log phase of NRB growth was extended to 84 hours. The growth rate increased to 6.3 × 10^6^ CFU per mL per hour compared with its growth behavior in glucose medium. During the initial 84 hours, 88.3% of nitrate was consumed at a rate of 23.0 mg (L h)^−1^ and the biomass reached 0.702 Abs on glycerol medium. In contrast, only 26.6% of nitrate was consumed at a rate of 7.4 mg (L h)^−1^ when CX3 grew on glucose medium. Low accumulation rates of biosurfactants (0.023 and 0.049 CMC h^−1^ on medium 1 and 2, respectively) was observed on both of the two mediums within 60 hours when the nutrients of nitrate and substrates were mainly assimilated into body cells. However, the production rate of biosurfactants increased to 0.65 CMC h^−1^ between the time range of 60 h and 156 h on medium 2 although a low rate of 0.032 CMC h^−1^ was observed on medium 1 during this period. Interestingly, the results indicated that high-speed yield of biosurfactants starts from the last stage of high rate of biomass accumulation and nitrate consumption (60 h). The surface tension of the CX3 culture broth was finally lowered to 29.6 mN m^−1^. Zhao *et al.*^33^ constructed rhamnolipid-producing recombinant strain *Pseudomonas stutzeri* Rhl by cloning the rhamnosyltransferase gene rhlABRI from *Pseudomonas aeruginosa* SQ6 into a facultative anaerobic denitrifying bacterial strain *Pseudomonas stutzeri* DQ1. They utilized glycerol as carbon source for *Pseudomonas stutzeri* Rhl and similar surface tension (30.6 mNm^−1^) was obtained through anaerobically produced rhamnolipid.

### 3.4 Characterization of NRB-produced biosurfactants

After the extraction, the final concentrated biosurfactant product by *P. stutzeri* CX3 was with a yellow-brown colour. It could lower the surface tension of distilled water from 72.2 mN m^−1^ to 30.4 mN m^−1^. The biosurfactant product was further characterized and the stability under various environmental conditions was also examined to facilitate its potential use in the fields.

#### 3.4.1 CMC of biosurfactants

CMC is an important indicator which determines the capability of biosurfactants to mobilize crude oil from contaminated soils or the sand-oil mixture into the aqueous biosurfactant solution. Low CMC is correlated with the high efficiency of the biosurfactant product.^55^ The CMC value of the isolated product was determined by measuring the surface tension of different concentrated solutions of the product with a tensiometer, and a sudden change in the surface tension was observed. A lower CMC value of 35 mg L^−1^ was derived for the extracted biosurfactant product. The product proved to be highly efficient when compared with the typical CMC values (10–230 mg L^−1^) of previously reported biosurfactants produced from different microbial sources.^56,57^

#### 3.4.2 TLC analysis

The lipid content in the biosurfactant product was determined through the general staining of iodine. TLC analysis also showed that light red spot on silica gel plates was generated when using ninhydrin as color developing reagent, suggesting that only a small part of lipopeptides existed in this biosurfactant product. In contrast, the evident yellow spot proved the abundant presence of glycolipids. Zhao *et al.*^33^ constructed an engineered strain *Pseudomonas stutzeri* Rhl and used it for heterologous production of Rhamnolipids under anaerobic conditions. Accordingly, the main components of glycolipid biosurfactants were most probable to be rhamnolipids. All the results indicated that the product was very likely to be a mixture of a small part of lipopeptides and a large part of glycolipids.

#### 3.4.3 Lipid and fatty acid analysis

TLC-FID analysis revealed that this biosurfactant product was a mixture of seven lipid components, which was dominated by the acetone mobile polar lipids and phospholipids. As shown in Table 2, 46.7% of the components were acetone mobile polar lipids (mainly glycolipids), accounting for nearly half the total lipids. Phospholipids, as the most abundant lipids in cell membranes and dominating constituent in our lipids matrix (31.9%), may be partially originated from cell debris co-precipitated with the biosurfactant product during the extraction process. The fact could also be inferred from the presence of small amounts of sterols in lipids (1%) as cholesterol is an important structural component of a phospholipid bilayer.^58^ Additionally, hydrocarbons, triacylglycerols and free fatty acids were also found in various amounts in the lipid mixture.

The lipid components determined by TLC-FID and fatty acid profiles by GC-FID of the biosurfactant

**Table:** 

Components	Composition (%)
Hydrocarbons	7.3
Ethyl ketones	0.7
Triacylglycerols	4.7
Free fatty acids	7.8
Sterols	1.0
Acetone mobile polar lipids	46.7
Phospholipids	31.9

**Table:** 

Fatty acids	Composition (%)	Fatty acids	Composition (%)
14:0	2.3	17:0	0.3
i15:0	2.0	17:1	3.3
a15:0	13.8	18:0	1.4
15:0	0.2	18:1w11	0.1
i16:0	5.5	18:1w9	1.0
16:0	15.8	18:1w7	20.2
16:1w11	0.3	18:1w6	0.2
16:1w7	11.7	18:1w5	0.2
16:1w5	11.2	18:2w6	1.2
i17:0	1.7	18:3w3	1.9
a17:0	5.3	20:1w9	0.1
Phytanic acid	0.2	22:0	0.02
Saturated	20.2	PUFA	3.1
MUFA	48.5	Bacterial	32.2

Further fatty acid profiles (Table 2) revealed that the fatty acids were mainly monounsaturated fatty acids (MUFA) and bacterial fatty acids (48.5% and 32.2%, respectively). Polyunsaturated fatty acids (PUFA) were found in very limited amount, accounting for only 3.1% of the total. The three most abundant fatty acids from the complex matrix were C18:1w7, C16:0 and a-C15:0, whereas the long-chain acids C16 and C18 contributed up to 70.9% of the total. This phenomenon can be attributed to their metabolic pathway during which the fatty acids were biosynthesized from the stepwise addition of two-carbon units derived from the building-block acetyl-coenzyme A (CoA) to a growing chain.^59^ During our experiments, the biosynthesis pathway is more relevant to the fatty acid compositions of products than the variations of carbon source. Similar results of fatty acid composition were obtained by Morita *et al.*^34^

#### 3.4.4 FT-IR analysis and microbe-mediated nitrate reduction effect on biosurfactant production

**Fig. 4:**
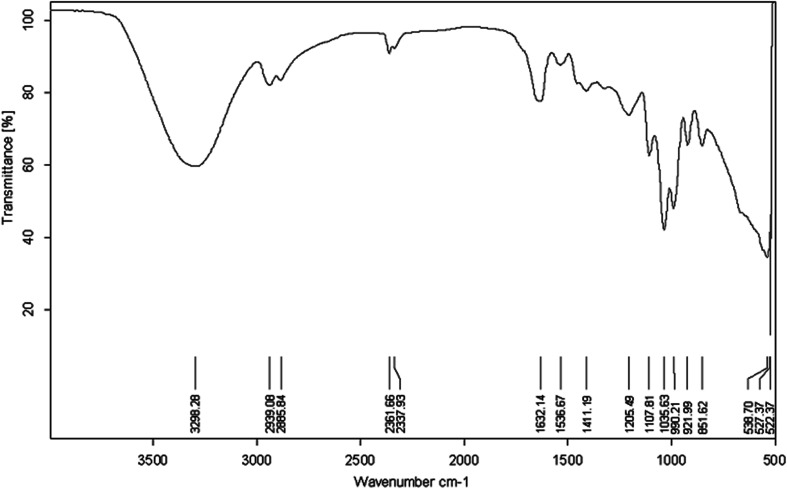
FT-IR transmittance spectrum of the extracted biosurfactant product generated by *Pseudomonas stutzeri* CX3 grown on glycerol medium.

The molecular composition of the biosurfactant product generated by *P. stutzeri* CX3 was evaluated by FT-IR and the FT-IR spectra were shown in Fig. 4. The broad absorbance peak centered around 3298 cm^−1^ indicated the presence of stretching OH bonds and N–H bonds of protein. Absorption around wave numbers 2939.08 and 2885.84 cm^−1^ were assigned to the symmetric C–H stretches of –CH_2_ and –CH_3_ groups of aliphatic chains. The aliphatic chains were also reflected from bending vibrations at 1411.19 cm^−1^. We also observed the protein-related bands the –C=O amide I (1632.14 cm^−1^) and –NH/–C=O combination of the amide II bands (1536.67 cm^−1^). However, just like the presence of phospholipids from lipid analysis results, it might be possible that the two bands at 1632.14 cm^−1^ and 1536.67 cm^−1^ resulted from polypeptides originated from cell debris co-precipitated with the biosurfactant product during the extraction process.^28^ The absorption peaks of 1035.63 and 1107.81 cm^−1^ were expected to be stretching C–O–C bonds, which indicated the presence of polysaccharide or polysaccharide-like substances in the biosurfactant product. The FTIR spectra provided strong evidence for the presence of glycolipids and lipopeptides in the isolated biosurfactant product.^60,61^

**Fig. 5:**
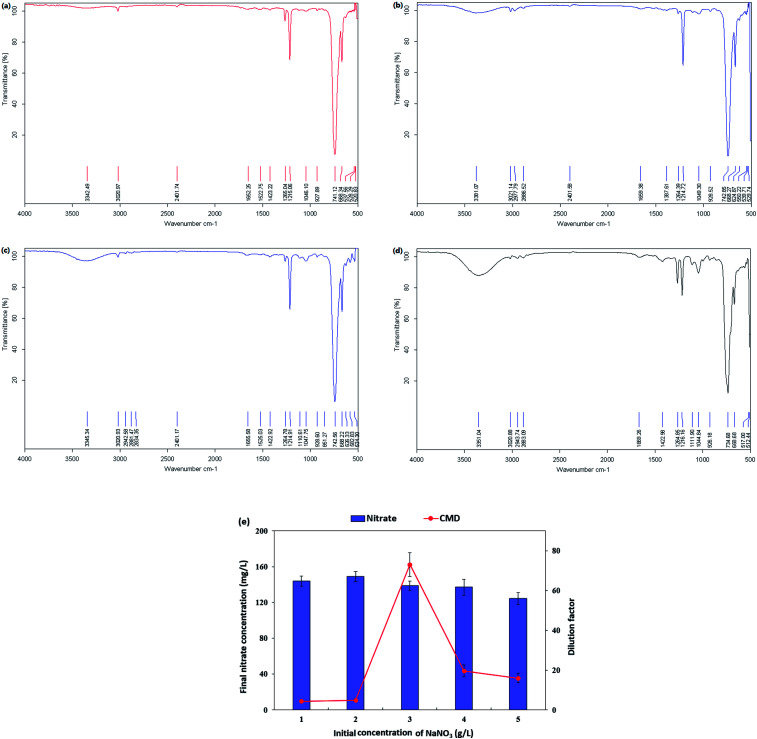
FT-IR transmittance spectrum of the extracted biosurfactant products, nitrate consumption and dilution factor of biosurfactant products generated by *Pseudomonas stutzeri* CX3 under various nitrate conditions. (a) NaNO_3_ at 1 g L^−1^; (b) NaNO_3_ at 2 g L^−1^; (c) NaNO_3_ at 4 g L^−1^; (d) NaNO_3_ at 5 g L^−1^; (e) nitrate consumption and dilution factor of biosurfactant products.

The nitrate-reducing capacity of by *P. stutzeri* CX3 was closely correlated with the amount, kind and component of the biosurfactant product. Fig. 5 showed the FT-IR transmittance spectrum of the extracted biosurfactant products, nitrate consumption and dilution factor of biosurfactant products generated by *P. stutzeri* CX3 under various initial nitrate conditions. It was revealed that the absorption peaks around 700 cm^−1^ and 1250 cm^−1^, which indicated the existence of –CH_2_ group and –C=O deformation vibrations, respectively,^60^ remained relatively stable under the four nitrate conditions. The absorption peaks around 3350 cm^−1^, which indicated the existence of stretching OH bonds and N–H bonds of protein, increased with the increased nitrate addition. This is attributed to the fact that sufficient nitrate addition promoted the bacterial growth and the generation of lipopeptides in the isolated biosurfactant products. However, excessive nitrate addition will inhibit the biosurfactant production. As indicated in Fig. 4 and 5e, NaNO_3_ concentrations at 3 g L^−1^ yielded the most abundant and diverse biosurfactant product. Interestingly, the final nitrate concentration all reached around 140 mg L^−1^ regardless of the initial concentrations. Nitrate concentration significantly affected the final proportion of glycolipids and lipopeptides in the isolated biosurfactant product and the associated productivity.

#### 3.4.5 Stability studies

**Fig. 6:**
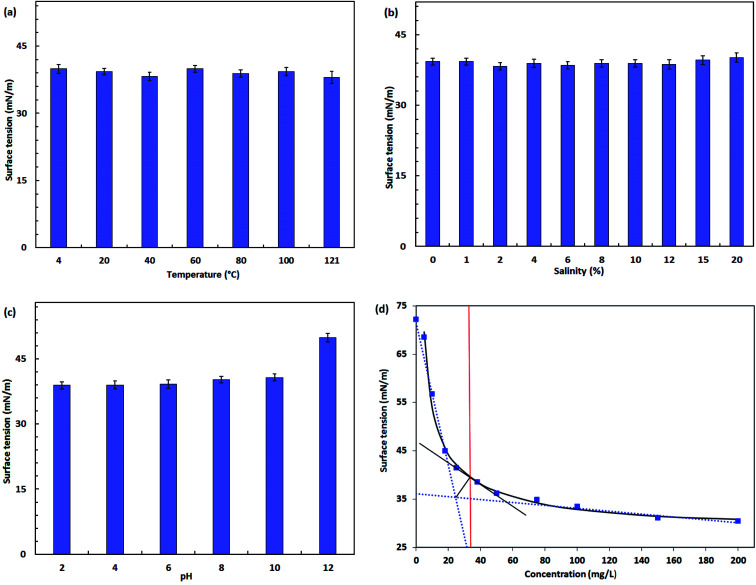
Stability of the biosurfactant product produced by *Pseudomonas stutzeri* CX3 under various environmental conditions. (a) Temperature; (b) salinity; (c) pH; (d) critical micelle concentration (CMC) of the biosurfactant at 35 mg L^−1^.

The applicability of biosurfactants in several fields is highly associated with their stability under various environmental conditions. Therefore, after the generation of biosurfactant product by *P. stutzeri* CX3, its stability was tested under a wide range of temperature, pH value and salinity while maintaining the biosurfactant concentrations at CMC value (35 mg L^−1^). From the results shown in Fig. 6, the biosurfactant activity was retained and insignificantly affected by most of the experimental settings, especially only small variations in surface tension values were determined under the wide range of temperatures between 0 and 121 °C and salinities between 0% and 20%. Biosurfactants are widely applied in petroleum, pharmaceutical, health care and food processing industries^62,63^ due to its robust stability and the findings indicated the potential use of our product in these areas. As indicated by Fig. 6a, biosurfactant solution achieved the lowest surface tension at a temperature around 40 °C, whereas heating up to 100 °C or even autoclaving (121 °C) caused no significant effect on its thermal stability. The results are accorded with the properties of rhamnolipids indicated by Abdel-Mawgoud *et al.*,^64^ which are main components of the biosurfactant product and maintain stable structure over the temperature settings. The extreme thermal stability was reported by Kiran *et al.*^65^ from *Brevibacterium aureum* MSA13 and Aparna *et al.*^60^ from *Pseudomonas* sp. 2B, which enables the biosurfactant product to be applied in a high-temperature reservoir.

From Fig. 6b, negligible changes were observed in the increased concentration of NaCl up to 20% (w/v). Electrolytes in the bulk solutions will shield the carboxylate groups of the rhamnolipid molecules, causing them to behave more like nonionic than anionic surfactants.^66^ Although a relatively lower surface tension was obtained at 2% NaCl concentration, the differences over the wide brine concentration levels of 0 to 20% were not significant (<5%). The steady performance of surface tension in salty waters allows the product to be potentially used in reservoir microbial enhanced oil recovery (MEOR) or environmental bioremediations under saline environments. For instance, Singh and Tripathi^67^ isolated a strain of *Pseudomonas stutzeri* from the formation water of an Indian coalbed and the isolate produced a copious amount of biosurfactant with the supplementation of coal. The biosurfactant with rhamnolipid nature showed great potential for *in situ* biotransformation of coal into methane and bioremediation of PAH-contaminated sites.

The surface activity of biosurfactant solution remained relatively stable between pH 2 and 10. A negative biosurfactant performance was observed at pH 12 in which the surface tension raised to 49.9 mN m^−1^. This detrimental effect is possibly caused by structure alteration of the biosurfactant product under extreme pH conditions. The polar head of anionic rhamnolipids was more negatively charged under more alkaline conditions.^38^ This is reflected by the fact of increased solubility of the product.

As amphiphilic molecules released extracellularly by microorganisms, biosurfactants are known to be beneficial for their producers in various ways. By promoting wetting, solubilization and emulsification of various types of organics, biosurfactants could increase the surface area between the oil and water phases, thereby increasing the bioavailability of entrapped oil in the porous media.^17^ Thus, biosurfactants could improve the nutrient conditions of NRB producers and enhance the competence of NRB towards SRB. Additionally, several biosurfactants proved to have antibiotic effects,^14^ which may have the potential to inhibit the growth of SRB. Moreover, biosurfactants were found to be important agents in the connection between microbial communities and biofilm formations,^68^ which may synergistically improve the resistance of the NRB producers to harsh environment. Therefore, the generated biosurfactant product, once injected into the soured reservoir externally, has great potential to assist NRB outcompeted SRB through these mechanisms. The effects of NRB produced biosurfactants in offshore reservoir NRB/SRB interactions, coupled with other environmental implications of this product, need to be further investigated.

## 4. Conclusions

In this study, the offshore petroleum-reservoir brines following nitrate/nitrite injection were used for the anaerobic screening of biosurfactant producing NRB. After periodic enrichment and sophisticated screening of the microorganisms, five typical denitrifying strains were isolated and found to be the species of *Pseudomonas stutzeri* according to their 16S rRNA sequencing results. The isolates were further screened for possible biosurfactant producers on glycerol and glucose media. The strain *P. stutzeri* CX3 was confirmed with biosurfactant production capacity through a series of biosurfactant characterization tests (*e.g.* drop collapsing test, parafilm test and surfaced tension determination). Better surface tension lowering ability was observed from the glycerol medium and the consumption of nitrate by NRB was found in positive correlation with bacterial growth and surface tension reduction. CX3 was selected to further demonstrate the performance of biosurfactant production through two production media over a 228 hour monitoring. The nitrate concentrations and surface tensions on the two media were both reduced to a relatively stable level within 84 hours during which OD_600_ reached relatively high levels as well over the period. The biosurfactant produced by *P. stutzeri* CX3 was defined with a CMC as low as 35 mg L^−1^, and further characterized by TLC, GC-FID and FT-IR analysis. The main components of the biosurfactant product were recognized as glycolipids. The biosurfactant product demonstrated stable performance during different environmental conditions with a wide range of temperature, pH values, and saline environment and have potential applications in environmental bioremediation, petroleum and other various industrial fields. The successful isolation and identification of biosurfactant producing nitrate-reducing bacterium from laborious screenings on offshore reservoir samples, coupled with subsequent biosurfactant characterization would provide new insight into NRB/SRB interactions for offshore reservoir souring control investigations.

## Conflicts of interest

The authors declare no conflict of interest.

## Supplementary Material

RA-008-C8RA03377C-s001
