# Supplementary material for: Isolation of nitrate-reducing bacteria from an offshore reservoir and the associated biosurfactant production
Source: RSC Adv. 2018 Jul 25;8(47):26596–609. doi: 10.1039/c8ra03377c (PMC9083026; doi:10.1039/c8ra03377c)
Supplement: RA-008-C8RA03377C-s001 [file RA-008-C8RA03377C-s001.pdf]

Electronic Supplementary Material (ESI) for RSC Advances.
This journal is © The Royal Society of Chemistry 2018

Supplementary Material

Isolation of nitrate-reducing bacteria from an offshore reservoir and the associated biosurfactant production

Fuqiang Fan[1], Baiyu Zhang[1*], Penny L. Morrill[2], Tahir Husain[1]

*Corresponding author: Baiyu Zhang: bzhang@mun.ca

Supplementary Figures

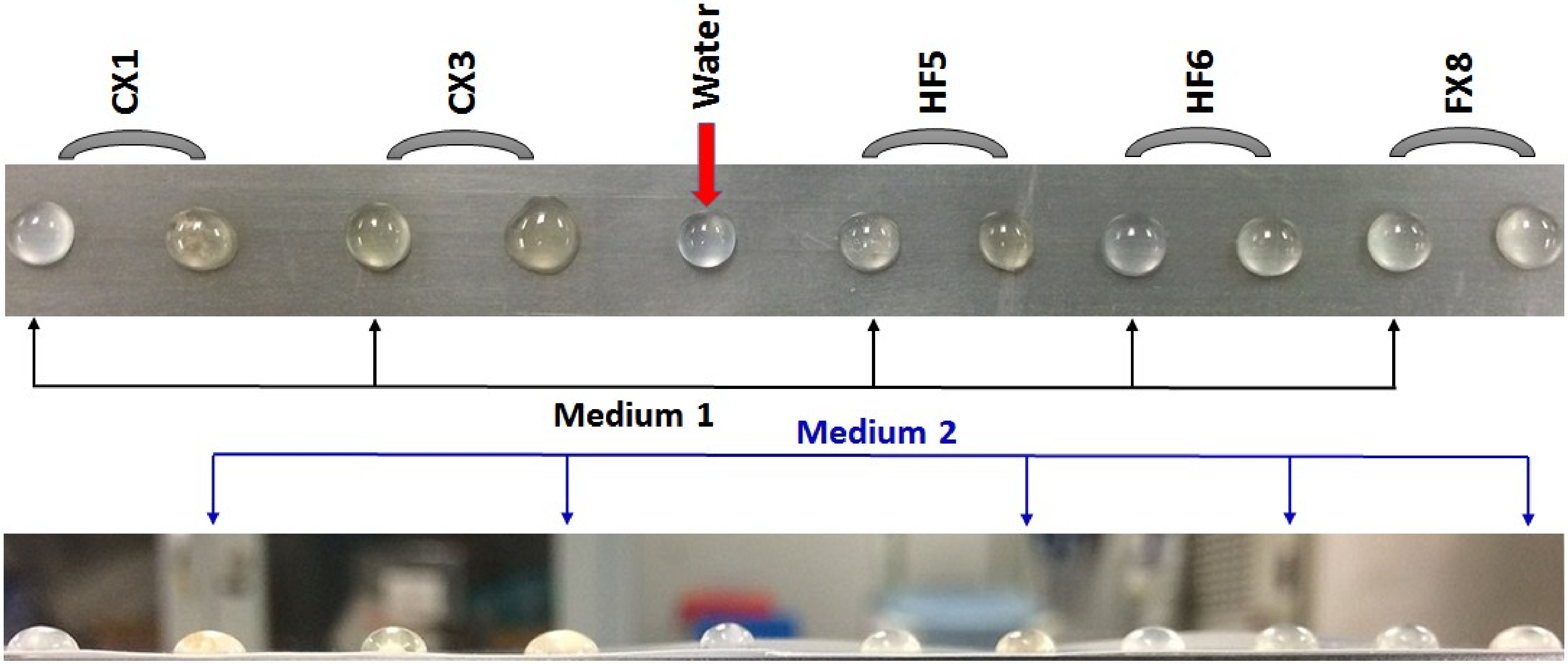

Figure S1 Secretion of amphipathic biosurfactant reduces the surface tension of the culture supernatant as indicated by the degree of flatness of droplet on parafilm.
